# Former Epidemics

**Published:** 1918-11

**Authors:** 


### Former Epidemics.

The wide-spread prevalence of influenza has started an investigation as to previous epidemics on the part of those who like to study such things. Capt. Howard J. Cole, who has made a wide study of pests and plagues gives this interesting information to those who have not taken the time to investigate for themselves:

"There have been seven other widespread epidemics of influenza in the United States and the effect has been comparatively the same in each instance and has been attended by the same panicky feeling on the part of the public. The first was in 1762 and the others in 1782, 1787, 1803, 1833, 1837 and in 1847. These epidemics seem to have originated in Asia and traveled westward through Europe and on to the United States.

"Scientific inquiry to ascertain the cause of this remarkable ailment has been conducted almost unceasingly for more than 100 years and many theories have been advanced. By some it has been held to depend upon some telluric and climatic conditions, but the occurrences of the disease ravages down through the century in all sorts of climate negatives this theory.

"Back in the Seventeenth century the Italians ascribed the mysterious malady to the influenza of the stars, and hence the name 'influenza,' by which it has subsequently been known.

"The best authorities show that during all the epidemics the disease asserted itself in the same manner—suddenly with all the characteristics of a severe cold. At first there are chills and rigors, then distressing headaches and tightness across the forehead, watering eyes, sneezing, coughing and disturbance of the digestive system.

"Never before in the history of the epidemics has the death toll been so heavy, but now the congestion is greater in the cities."

